# Rapid warming in Tibet, China: public perception, response and coping resources in urban Lhasa

**DOI:** 10.1186/1476-069X-12-71

**Published:** 2013-08-27

**Authors:** Li Bai, Cirendunzhu, Pengcuociren, Dawa, Alistair Woodward, Xiaobo Liu, Baimaciwang, Dazhen, Shaowei Sang, Fangjun Wan, Lin Zhou, Junfang Xu, Xiaolu Li, Haixia Wu, Baorong Yu, Xiraoruodeng, Qiyong Liu

**Affiliations:** 1 Collaborative Innovation Center for Diagnosis and Treatment of Infectious Diseases, State Key Laboratory for Infectious Diseases Prevention and Control, National Institute for Communicable Disease Control and Prevention, Chinese Center for Disease Control and Prevention, 155 Changbai Road, Changping District, Beijing 102206, P. R. China; 2 Tibet Center for Disease Control and Prevention, 21 Linkuo North Road, Lhasa 850000, Tibet, P. R; 3 School of Population Health, University of Auckland, Private Bag 92019, Auckland 1142, New Zealand; 4 Center for Health Management and Policy, Shandong University, 44 WenhuaXi Road, JiNan 250014, Shangdong, P. R. China; 5 Climate Change and Health Center, Shandong University, 44 WenHua Road, JiNan 250012, Shangdong, P. R. China

**Keywords:** Climate change, Global warming, Perceptions, Human health, Tibet, Vulnerability

## Abstract

**Background:**

Tibet, average altitude more than 4,000 meters, is warming faster than anywhere else in China. The increase in temperatures may aggravate existing health problems and lead to the emergence of new risks. However, there are no actions being taken at present to protect population health due to limited understanding about the range and magnitude of health effects of climate change.

**Methods:**

The study was a cross-sectional survey of 619 respondents from urban Lhasa, Tibet in August 2012 with the aim to investigate public perceptions of risk, heat experiences, and coping resources.

**Results:**

Respondents are aware of the warming that has occurred in Lhasa in recent years. Over 78% reported that rising temperature is either a “very” or “somewhat” serious threat to their own health, and nearly 40% reported they had experienced heat-related symptoms. Sex, age, education and income influenced perceived risks, health status, and heat experience. The vast majority of respondents reported that they had altered their behaviour on hot summer days. Bakuo, a sub-district at the city center, is considered especially vulnerable to heat because of sparse vegetation, high population density, poor dwelling conditions and a high proportion of low-income population. However, neighborhood social ties were stronger in Bakuo than other study locations.

**Conclusions:**

The study suggests that actions are needed now to minimize downside effects of rapid warming in Tibet, because of increasing human exposure to high temperatures and uneven distribution of the resources needed to cope.

## Background

Heat is a threat to human health in many parts of the world [1,2]. The Fourth Assessment Report of the Intergovernmental Panel on Climate Change (IPCC) points out that climate change is already exacerbating the global burden of disease and premature deaths and the impact of high temperatures is expected to increase in the future [3]. A large amount of research has investigated the effects of heat on public health and vulnerability to heat [4]. Most of these studies were conducted in developed countries and less information is available from developing countries though these are considered to be most vulnerable to climate change and climate variability.

Tibet of China, lying at an average altitude of more than 4,000 meters, covers more than 1,228,400 square kilometers, and accounts for one eighth of China’s total land mass. It has been called “the third pole of the world” and “the Asian water tower” [5]. In recent years, Tibet has faced a number of environmental challenges, including changes in the climate [5-7]. Average temperatures in Tibet have been rising 0.32°C every decade since the 1960s, four times faster than elsewhere in China and twice the global average increase [8]. Winter temperatures in Tibet have been increasing at a faster rate than any other inland areas of China, between 0.29°C ~ 1.04°C every decade [9]. Indeed, the Tibetan plateau, a region which contributes minimal carbon dioxide emissions, is likely to be one of the areas most sensitive to global warming [8]. Changes are evident in the capital city, Lhasa. In 2009, on the hottest summer day, the maximum temperature in Lhasa reached 30.4°C. Before this, the highest record was 29.9°C in 1971. An enhanced urban heat island effect has been observed in Lhasa, especially in winter [10]. If warming continues it is projected that Tibetans and millions of people in western China will be exposed to more frequent severe weather events [8].

Previous research has suggested that an individual’s perceived risk is closely related to daily mitigating actions and adaptive behaviour changes [11]. However, despite the increase of epidemiological studies of heat and health, little work has been done on how the public perceives heat [11-15], particularly in developing regions. To our knowledge, no research to date has been conducted in Tibet to examine public views of the health impacts of rising temperatures. The goal of the study was to explore public perceptions, experiences of heat-related symptoms and behaviour changes in four urban sub-districts in Lhasa. The study also evaluated coping resources in the four study locations, based on dwelling conditions, the prevalence of home air conditioning and relationships with neighbours.

## Methods

### Settings

**Figure 1:**
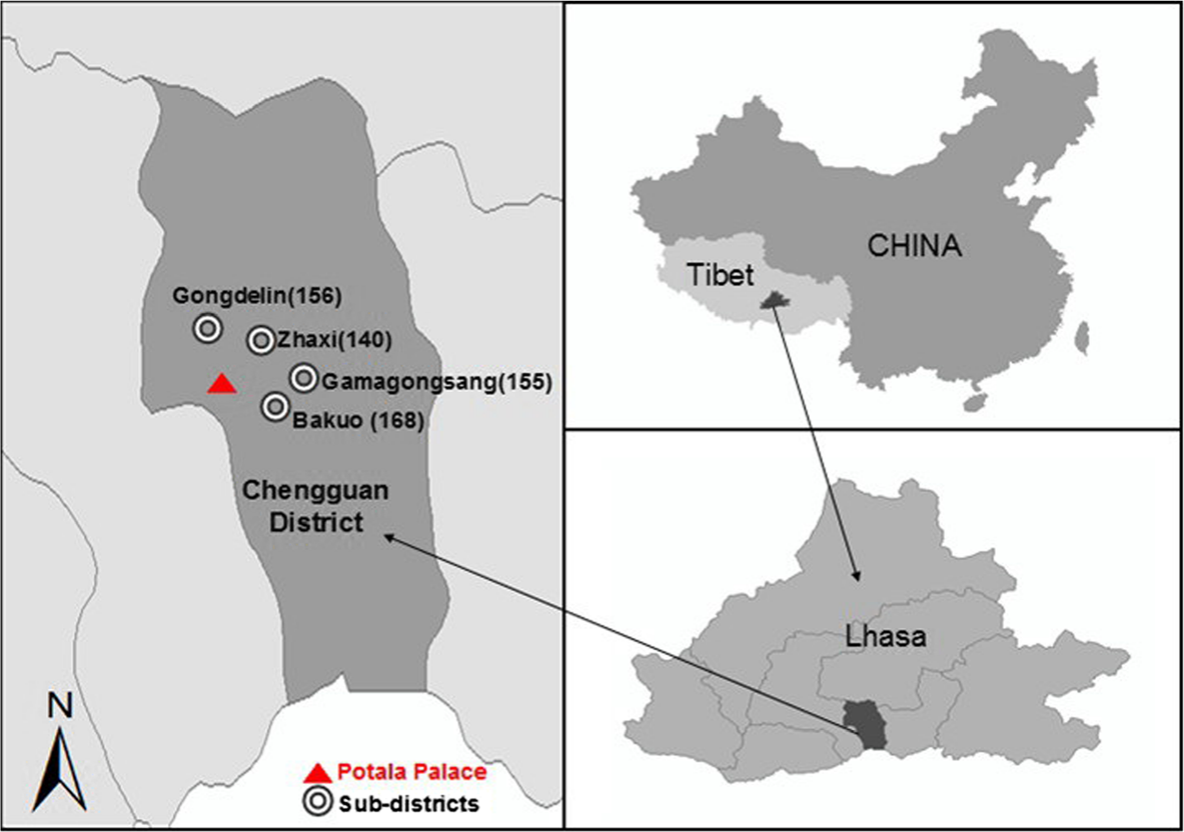
**The four urban sub-districts into this research.** The *numbers in parentheses* represent the sample sizes for the survey work.

The data presented in this paper were obtained by questionnaire surveys from 16 through 19 August, 2012 across the urban area of Chengguan district, which is the one and only municipal district of Lhasa City. We selected purposely four of six urban sub-districts (Gongdelin, Gamagongsang, Zhaxi and Bakuo) (Figure 1) in Chengguan district to ensure good geographical coverage of urban Lhasa and obtain data that best present the diverse demographic and socio-economic characteristics of local urban population. The four sub-districts (Table 1) are composed of 20 communities and the socio-demographic pattern of communities within the same sub-districts are similar. The survey was carried out in the 20 Community Committees, which are normally located at the geographic centers of the communities and are well known among local residents.

**Table 1:** Population density of four study locations provided by Sub-district Offices.

	**Gongdelin**	**Gamagongsang**	**Zhaxi**	**Bakuo**
Total population*	31492	26165	44775	24650
Area (km^2^)	4.1	3.2	6.0	2.7
Population/km^2^	7757	8177	7462	9129

* Total population comprises of both resident population and mobile population.

**Figure 2:**
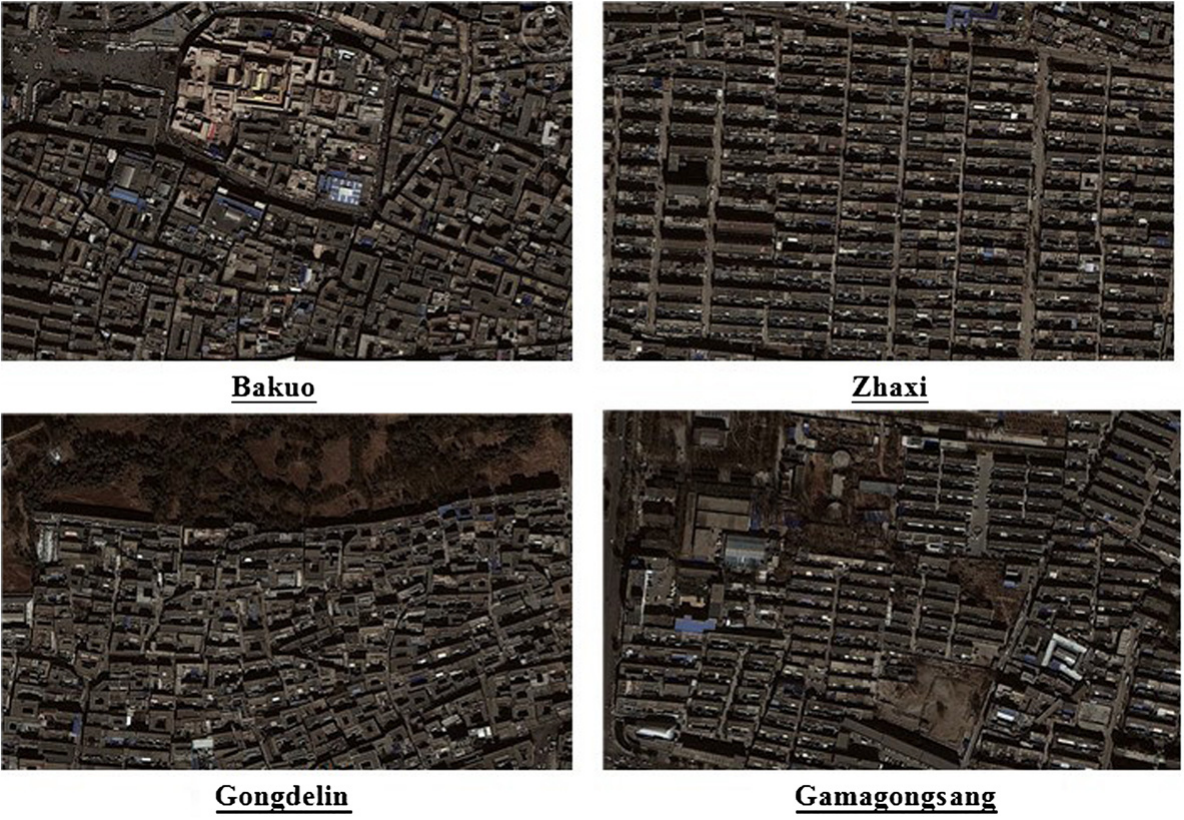
Aerial photographs of four sub-districts in urban Lhasa (Google Earth).

*Bakuo* is in the business center of the urban core and is often called the “old town”. One of the most famous temples in Tibet, Dazhao Temple is located in this sub-district and surrounded by many business shops, restaurants, hostels and residential buildings. It is the most popular, noisy and crowded place in urban Lhasa. Residents tend to have low educational level, low income, small living spaces and poor dwelling conditions. As seen from the aerial photograph of *Bakuo* (Figure 2), there is a circular street around Dazhao Temple which is frequently crowded with local Buddhists and tourists. The surrounding tube-shaped buildings are mainly residential apartments and hostels, but the first floors of these buildings contain shops and restaurants. There is very little open or green public space within the sub-district. *Gongdelin* is approximately 3 km from *Bakuo* and on the north borders the “Lalu Wetland”, known as the “Lung of Lhasa” for its significant role in providing oxygen and cleaning air in Lhasa. This is the highest natural wetland in the world, covering 12.2 square kilometers. In addition, a large green park near the Potala Palace within this sub-district provides an attractive shaded area in summer. Residents used to live on yak herding at Lalu Wetland, but now mainly earn money from renting houses and running inns because yak herding is forbidden in order to protect the wetland. Residents are relatively wealthy and mainly live in self-built single family houses with small yards. *Zhaxi* and *Gamagongsang* are sub-districts with limited infrastructure such as drainage system, roads and fire escape. Most residents do not have a toilet at home and neither of the two sub-districts has a park. Compared to *Zhaxi,* there is more open and green public space in *Gamagongsang*.

### Data collection and analysis

To measure perceptions of climatic warming and responses among the urban population in Lhasa, we developed a questionnaire based on previous studies of climate change and human health risks [11-15]. In order to localize the survey and make it culturally acceptable, the questionnaire was developed in consultation with public health officials of Tibet CDC (Center for Disease Control and Prevention). All questions in the survey were close-ended questions using different Likert-type items or other categorial items such as “Yes”, “No”, “Don’t know” or “Don’t remember”.

Section ‘A’ asked several questions about heat perceptions including whether the weather in Lhasa is getting warm and will become warmer in future, how large a health risk due to climatic warming was perceived to be, for human beings in general and for themselves, which natural disasters were biggest threat to themselves and how dangerous future warming was perceived. Previous epidemiologic studies have linked heat-related illness to chronic conditions, particularly pre-existing cardiovascular and respiratory illness. Section ‘B’ asked questions on health status of the respondents and experiences of heat. A question was included on what types of information would be helpful to cope with heat-related illness. Section ‘C’ included questions on how people modify their behavior on hot days, and this was followed by a series of questions regarding economic and social resources for dealing with heat covering dwelling quality, air conditioning and/or a fan at home, and neighborhood social ties, as reported in a previous heat vulnerability study [16]. The final section ‘E’ sought socio-demographic information from the respondent.

We pre-tested the instrument to ensure all questions were clear and understandable. More than forty field interviewers (including our research team members, local public health staff and staff working with sub-district offices and community committees) were then trained by the first author and corresponding author. All local interviewees were able to speak both Mandarin and Tibetan. Training lasted five hours and included an item-by-item review of the questionnaire, survey methods, communication skills and social and cultural sensitivity. Interviewees practised the survey scripts with a paired partner. The first author and corresponding author reviewed each interviewee’s survey, pointed out inappropriate methods, manners and choice of words, and inspected the quality of each survey script.

Surveys were administered to residents older than 18 years who lived in the four urban sub-districts all year around. After consulting with local Community Committees, we realized that it was not possible to recruit participants by random sampling due to local cultural sensitivities. Also, the study was carried out during daylight hours because it is not culturally acceptable by Tibetans residents to conduct interviews at other times. Three days before the surveys started, the staff of each Community Committee informed all residents living in the community about the purpose, period and location of the survey and encouraged them to participate. Those who agreed to be interviewed then came to the Community Committee to complete the survey during the study period.

In order to avoid possible bias, we developed face-to-face questionnaire surveys which were filled out by trained interviewees after asking questions one by one. In order to improve representativeness, the age ratio of the Sixth Census of Lhasa in 2010 was used as the reference in selecting the number in each age group. We obtained ethical approval from the Ethical Review Committee of Chinese Center for Disease Control and Prevention (No. 201214). Written informed content was obtained from all participants prior to survey and all data obtained were anonymized. The data were analyzed by EpiData 3.1 and SPSS18.0. A χ2 test was used to calculate the difference between groups. A stepwise multivariate regression was also applied to examine variables which were significantly associated with having had a heat-related illness in this summer.

## Results

### Demographic information

Six hundred and nineteen completed questionnaires (377 female, 242 male) were collected (Table 2). The high proportion of female respondents is attributed to the fact that the majority of males go out for work in the daytime, while females tend to stay home. In addition, females may be more likely to participate and complete the surveys. The age distribution of respondents is similar to that of all adults in the 2010 Census. Ethnically, 97.6% of the sample were Tibetan, which is higher than the proportion in the overall population (88.0%). Han Chinese and other ethnic groups in Lhasa are mostly young immigrants from other parts of China who have come to Tibet for jobs and thus may be less available to participate in surveys during daytime. In terms of education, 24.2% of respondents were illiterate and the majority of this group was in older age groups. Monthly income of participants varied across the four sub-districts. Low-income respondents (<1000 Yuan per month), who mostly lived on Basic Living Allowances from local government or in temporary jobs, accounted for 60.1% of the sample in Bakuo, 45.2% in Gamagongsang, 35.9% in Gongdelin and 24.3% in Zhaxi. Only 58 respondents indicated that they lived alone at present, and of this number, only 10 were aged 65 and above.

**Table 2:** Demographic characteristics of respondents

		**Total(N = 619)**	
		**n**	**%**
**Sex**	Female	377	60.9
	Male	242	39.1
**Age (yr)**	18-29	93	15.0
	30-41	160	25.9
	42-53	150	24.3
	54-65	126	20.4
	>65	89	14.4
**Ethnic**	Tibetan	604	97.6
	Han Chinese	11	1.8
	Others	4	0.6
**Education**	Illiteracy	150	24.2
	Primary school	216	34.9
	High school	204	33.0
	College and above	49	7.9
**Monthly income (RMB)**	<1000	261	42.2
	1000-2000	168	27.1
	2000-3000	78	12.6
	3000-4000	39	6.3
	4000-5000	21	3.4
	>5000	24	3.9
	Don’t want to say	28	4.5
**Living alone**	Yes	58	9.4
	No	561	90.6

### Perceived risk

**Figure 3:**
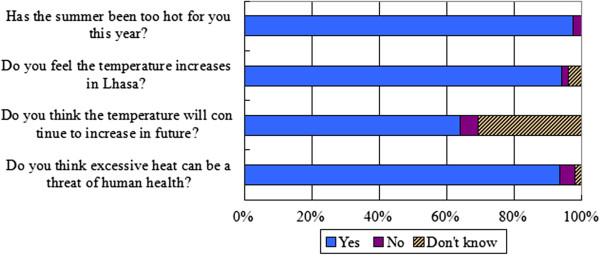
Responses to several survey questions regarding perceived risks of the climactic warming in Lhasa.

The overwhelming majority of respondents reported climatic warming in Lhasa is already happening and poses a threat to public health (Figure 3). Over 90 percent reported the summer is very hot and oppressive to them and believed that Lhasa has experienced rising temperatures in the past few decades. Approximately 65 percent believed the climate of Lhasa would become unbearably warmer in the near future. We found widespread agreement (92% of participants) that rising temperatures impact directly or indirectly on human health. Moreover, 78% believe that the rising temperature is either a “very” or “somewhat” serious threat to their own health or daily lives, while only 3% perceived they were not sensitive to temperature increase. The responses are similar across four sub-districts, but varied among different demographic groups of the sample. Females and older respondents were more likely to report that higher temperatures were dangerous. Interestingly, over 40% of high-income respondents (monthly income >5000 yuan) felt the heat was very dangerous to them, while only 18% of low-income people (monthly income <1000 yuan) gave the same answer.

To the question “how worried would you be if you hear about an excessive heat warning issue in Lhasa in the near future?” 23% reported that they would be very worried, and 61% reported they would be somewhat worried. Only 3% would be not worried at all. Not surprisingly, those who felt the temperature increase was very dangerous to them were more likely to report that they would be very worried about future heat warnings than those who did not believe they were in danger (47% vs. 5%). Respondents in Bakuo sub-district were slightly more likely than those in the other three sub-districts to report that they were ‘very’ or ‘somewhat’ worried about a heat warning in Lhasa. Lastly, participants were provided with several health-related risks and asked to select the biggest threat to them. Although the results indicated a wide recognition of climatic warming as a risk to human health among respondents, few of them (5%) believed extreme heat was the biggest threat of all. Over half reported that they were most fearful of natural disasters such as earthquakes. Air pollution, fires and traffic accidents were named by 17%, 14% and 8% respectively. Once again, the response varied among different income groups. The overwhelming majority of low income respondents were more likely to report that the natural disasters were the biggest threat, while those with relatively high incomes tended to select other risks apart from the natural disasters.

### Health status and heat-related condition

Respondents were asked about their health status, history of chronic illness and health-risk behaviors. Overall, 62% reported that they were ‘healthy’ and 32.3% felt their health status were ‘so so’, and the rest of them (5.7%) considered themselves having bad health. Thirty six per cent of the sample reported they were affected by chronic diseases. This group was more likely to report their health status was fair or bad and to perceive that heat was dangerous to them. Results differed across four sub-districts (p = 0.011), with the sample from Zhaxi (39.9%) reporting more pre-existing medical conditions than from others (Gongdelin 28.6%, Gamagongsang 29.8% and Bakuo 36.3%), mainly due to the higher proportion of elderly respondents in that sub-district. Chronic conditions included high blood pressure (47%), cardiovascular diseases (32%), respiratory diseases (18%), diabetes (3%) and cancer (1%). Moreover, 58.3% reported that they had been admitted to a hospital or taken to the emergency room because of the illness. Only 2.4% reported they drank liquor every day, and 15.7% smoked daily.

**Figure 4:**
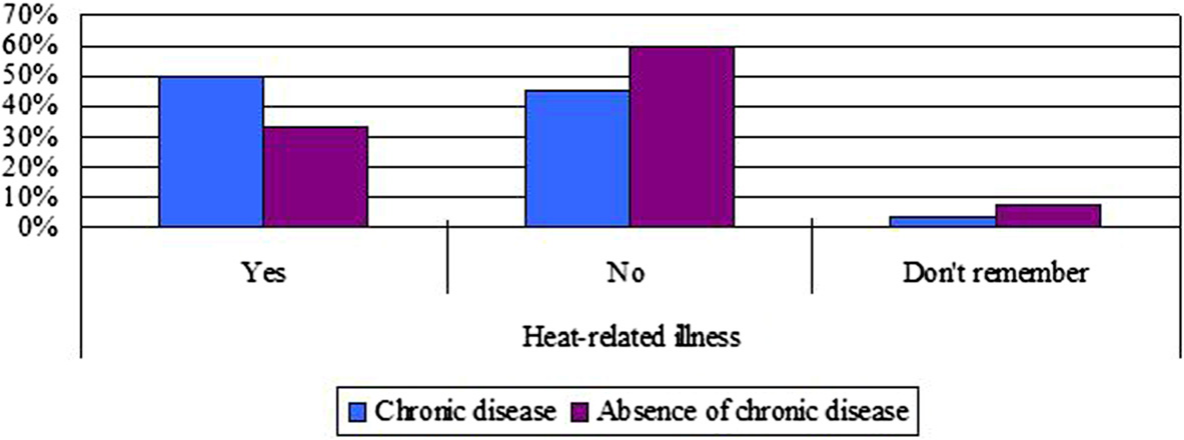
Heat-related illness by chronic conditions.

**Figure 5:**
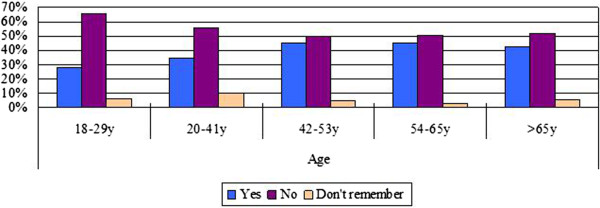
Heat-related illness by age groups.

Participants were asked directly whether they experienced any heat-related symptoms (including dizziness, tiredness, irritability, thirst, loss of appetite, headaches, nausea, vomiting, muscle weakness) during this summer, and 39.6% reported they experienced at least one of these symptoms. Older respondents, those with self-reported poor health, those with chronic diseases and those living alone were more likely to report they had been sick or physically uncomfortable because of the heat (Figures 4 and 5). Respondents in the 42–53 (27.5%) and 54–65 (23.8%) age groups reported higher rates of sickness (p = 0.047) than those 65 and older (15.6%). However, in a multivariate regression analysis, we found the only factor significantly associated with having had a heat-related illness was a history of chronic disease (Table 3). Among those with chronic conditions, 63% indicated that their symptoms had been aggravated because of the heat during this summer.

**Table 3:** Stepwise multivariate regression

**Model**	**Unstandardized Coefficients**		**Standardized Coefficients**	**t**	**Sig**
	**B**	***Std. Error***	***Beta***		
1(Constant)	1.324	0.083		15.863	0.000
Having chronic diseases	0.210	0.049	0.170	4.285	0.000

Dep. Var. = Having had a heat-related illness in this summer.

When participants were asked about the type of climate change and health information they may want to receive, most participants (72.5%) wanted to know about heat-related symptoms and illnesses, followed by 62.5% who wanted to know how to reduce the risks of heat-related illness, 56.4% who wanted to know what to do if you or others have any symptoms and 36.8% who wanted to know how does temperature increase affect human health. Very few respondents (1.3%) reported that they did not want to receive any information.

### Behaviour change

**Figure 6:**
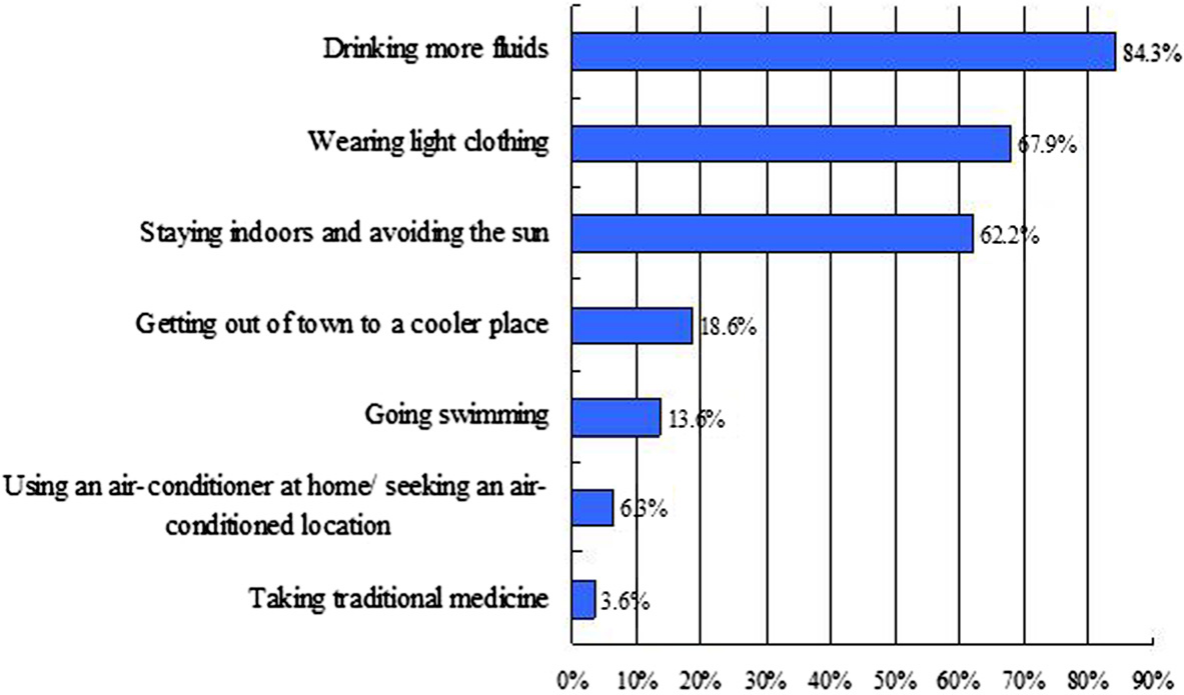
Options taken by respondents who reported they altered their behaviour in hot days.

The great majority of respondents (93.9%) reported that they had altered their behaviour on hot summer days. The percentage varied significantly (p = 0.027) across the four study sub-districts, ranging from 89% in Gamagongsang to 97% in Gongdelin. Of those that modified their behaviour, the most common measures cited were drinking more fluids, wearing light clothing and simply staying indoors and avoiding the sun (Figure 6). The responses varied somewhat among different demographic groups. Low-income persons were slightly more likely than high income respondents to drink more fluids, possibly explained by the prevalence of physical labour and outdoor activities among poor people. Young and middle aged respondents were more likely than the elderly to report they changed into light clothes on hot days (The older generation in Lhasa tends to maintain the traditional clothing habits of Tibetan long robes). Women and the elderly were more likely than the young to stay indoors. Other means cited by the respondents included getting out of town to a cooler place, going swimming, using an air-conditioner at home or seeking an air-conditioned location but few (3.6%) indicated they took traditional medicine to prevent heat-related illness.

Of those that responded that they did nothing in particular (6.1%) on excessively hot days, reasons included “the heat does not affect me” (n = 29), “don’t know what to do” (n = 10), “too much troubles” (n = 6) and “financial considerations” (n = 2). Behavior changes among participants on days when it was hot were associated with their perceived risk. Those who felt the rising temperature was not dangerous to them at all were more likely not to modify their behavior on hot days. Similarly, few persons who would be not at all worried about the heat warnings changed their behavior.

### Coping resources

Information was collected on dwelling conditions, using an air-conditioner/fan at home and social ties in the neighborhood (Table 4). In Bakuo, there are fewer self-built single family homes, and more old dwellings (built before 1980), multi-storied buildings with small rooms, and public rental apartments for low income individuals or households. We found an association between the dwelling conditions and heat-related illness, which was reported more commonly by respondents who live in apartments built before 1990 (47.1%, p = 0.016). Residents in Bakuo may be at higher risk of heat-related illness due to relatively poor living conditions and confined living spaces.

**Table 4:** Dwelling conditions of four sub-districts [%(n)]

		**Gongdelin**	**Gamagongsang**	**Zhaxi**	**Bakuo**	**Total**
		**(n = 156)**	**(n = 155)**	**(n = 140)**	**(n = 168)**	**(N = 619)**
Construction date	Before 1980	2.6 (4)	2.6 (4)	4.3 (6)	11.9 (20)	5.5 (34)
	1980-1989	15.4 (24)	11.6 (18)	38.6 (54)	11.9 (20)	18.7 (116)
	1990-1999	39.7 (62)	29.1 (45)	20.7 (29)	28.6 (48)	29.7 (184)
	2000 and after	28.8 (45)	38.2 (59)	19.3 (27)	36.3 (61)	31.0 (192)
	Don’t know	13.5 (21)	18.7 (29)	17.1 (24)	11.3 (19)	15.0 (93)
Structure type	Single family House	42.4 (66)	34.2 (53)	35.7 (50)	3.6 (6)	28.6 (177)
	Apartment	39.7 (62)	46.4 (72)	52.9 (74)	89.3 (150)	57.4 (355)
	Bungalow	17.9 (28)	19.4 (31)	11.4 (16)	7.1 (12)	14.0 (87)
Dwelling type	Self-build	44.2 (69)	34.8 (54)	27.1 (38)	1.2 (2)	26.3 (163)
	Purchase	23.7 (37)	36.8 (57)	60.0 (84)	33.3 (56)	37.8 (234)
	Public rental housing	21.2 (33)	13.6 (21)	5.7 (8)	61.9 (104)	26.9 (166)
	Private rental	10.9 (17)	14.8 (23)	7.2 (10)	3.6 (6)	9.0 (56)
Number of room	One	4.5 (7)	14.2 (22)	5.7 (8)	11.3 (19)	9.0 (56)
	Two	24.4 (38)	11.0 (17)	20.0 (28)	58.3 (98)	29.2 (181)
	Three	26.9 (42)	23.8 (37)	27.9 (39)	20.8 (35)	24.7 (153)
	Four and more	44.2 (69)	51.0 (79)	46.4 (65)	9.5 (16)	37.0 (229)
Air-conditioner in home	Yes	10.3 (16)	12.3 (19)	7.9 (11)	4.8 (8)	8.7 (54)
	No	89.7 (140)	87.7 (136)	92.1 (129)	95.2 (160)	91.3 (565)
Fan in home	Yes	13.5 (21)	19.4 (30)	18.6 (26)	20.8 (35)	18.1 (112)
	No	86.5 (135)	80.6 (125)	81.4 (114)	79.2 (133)	81.9 (507)

As expected, cooling devices are not common in Lhasa: only 8.7% of participants had air-conditioning at home, 18.1% reported they had a fan and about 6% had both. The percentage of home air conditioning ranged from 4.8% (Bakuo) to 12.3% (Gamagongsang). There were significant differences between income segments of the sample (p < 0.05), with a higher rate of air conditioning at homes of the upper income respondents (>4000 yuan/month, 13.5%). Among those not having home air conditioning, we asked whether they plan to install such a unit. Only one third reported that they might do so, if the temperature continues to rise in the future. Although air conditioning and other cooling devices are generally considered as having a strong protective effect during excessively hot days, we did not find an association between heat-related illness and the presence of air conditioning (p = 0.651) or a fan (p = 0.067) in one’s home (possibly because those who had air conditioners installed seldom turned them on) (Table 5).

**Table 5:** Heat-related illness and availability of air conditioning and fan at home

		**Has had heat-related illness during this summer (row percentage)**			**Total**
		**Yes**	**No**	**Don’t remember**	
**AC at home**	**Yes**	24 (44.2)	26 (48.1)	4 (7.4)	54 (100.0)
	**No**	220 (38.9)	309 (4.7)	36 (6.4)	565 (100.0)
	**Total**	244	335	40	619
**Fan at home**	**Yes**	55 (49.1)	50 (44.6)	7 (6.3)	112 (100.0)
	**No**	189 (37.3)	285 (56.8)	33 (6.5)	507 (100.0)
	**Total**	244	335	40	619

**Figure 7:**
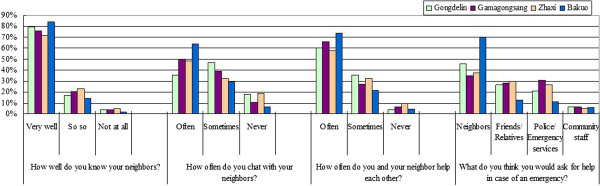
Responses to four questions of measuring social ties among sub-districts.

Strong community networks were evident in this study (Figure 7). The majority of respondents reported that they not only know their neighbors very well, but they often visited and helped each other. Moreover, nearly half of the respondents indicated that they would initially ask for help from their neighbors if an emergency occurred. Once again, a significant difference (p < 0.05) were observed, with even stronger neighborhood social ties in Bakuo than the other three locations (Figure 7). As expected, responses also varied across populations with different demographic and living characteristics. For example, the lowest percentage of seeking help from neighbors for an emergency was observed among those who have a college degree (31.5%, p = 0.001). Those living in private rental dwellings reported less contact with their neighbors. Therefore, though strong social ties are seen in Lhasa, particularly in Bakuo, there still some groups of the population that are less engaged, such as those with higher socio-economic status and the relatively mobile population (mostly Han Chinese and other non-Tibetan minorities) living in private rental dwellings.

## Discussion

This study is the first in Tibet to investigate public perceptions, experience, behaviour and coping resources regarding high temperatures and future climate change. The findings will inform subsequent quantitative studies including a map-based human health risk assessment and management plan. Overall, we found widespread awareness in the local population of rising temperatures and their effects on health. Some segments of the population are likely to be at greater risk due to environmental differences, demographic factors and limited coping resources.

Previous studies of this kind have been undertaken mainly in developed countries including the USA [11,12,15,17,18], Australia [19,20], and Canada [12,13]. Such studies can provide important information to policy makers and assist in developing and promoting practical adaptation measures. Our study has added a valuable dimension to the existing literature as little has been written previously about perceptions in relation to climate change in developing countries [14,21]. In many developed country studies there are high levels of knowledge and awareness about climate change [13] and heat warnings [11,15]. In Tibet, despite the lack of a heat plan or any educational program, we found that survey respondents were well aware of the rise in temperatures that has occurred and the effects this might have on health. This is consistent with another study conducted in Bangladesh, a developing country which has been identified as one of the most vulnerable to climate change [14]. In Bangladesh there was widespread awareness of global warming among respondents who were mostly rural people with little formal education. We surmise that people in low-income settings may be more likely to regard themself as vulnerable to heat than those in developed countries who live in more comfortable circumstances, are protected by advanced public health infrastructure and are informed via pervasive media coverage. If this is indeed the case, then there is both a need and an opportunity to introduce community-based heat adaption program in regions such as Tibet.

In our study, age and health status were found to be good indicators of perceived risk to heat. Older respondents, along with people with pre-existing chronic diseases saw themselves at greater risk from heat. Those with high incomes were also more likely to consider themselves to be sensitive to the heat, although it is low income groups who in fact are likely to be more vulnerable because of limited coping resources and access to information [16]. However, perception of risk does not mean that rising temperatures are the issue that most concerns people. Earthquakes were named as the biggest threat by most respondents which is understandable given local circumstances. In China’s recent history earthquakes have killed hundreds of thousands of people, and these tragedies have made a big impact on the Chinese population. On the other hand, in Phoenix, already one of the hottest cities in the US, over half of respondents treated increasing heat as the biggest threat, and fewer than 10% of them named earthquakes [11].

Previous papers addressing the effects of heat on health have concentrated on persons aged 65 years and over [22,23], and other age-groups have received much less attention. Our findings suggest that middle aged persons in Lhasa may be significantly affected the high temperatures: self-reported heat-related illness was most common in persons aged 42–65. There may be two reasons: this is an age group commonly affected by chronic medical conditions particularly cardiovascular diseases; and, unlike the elderly, most middle age people are still at work and thus may be exposed to more outdoor heat. Our finding is consistent with a model-based quantitative study which reported a marked effect of heat on mortality among person aged 45–64 [24]. The finding that people with chronic diseases reported a higher incidence of heat-related illness than those who do not have chronic medical conditions is in agreement with results from a recent Canadian survey [13]. Over 60% of respondents in our survey with chronic conditions felt their symptoms had been worsened because of the heat. In Tibet, there are unique environmental factors such as extreme altitude and low oxygen pressure that create special challenges to the health of local people. Chronic mountain sickness (CMS) is common, and is marked by hypoxia, high red cell count, and associated heart diseases [25-27], all of which reduce heat tolerance and make Tibetan people even more vulnerable to the rising temperatures forecast under climate change.

A most surprising result in this study is that the vast majority of respondents reported modifying their behaviour because of high summer temperatures. This is totally contrary to a previous study in North America suggesting that few people altered their behaviour during heat events, even if a heat warning or a heat advisory was issued [15]. We further observed that the widespread behaviour changes may exist on a common sense basis since measures most cited by respondents to deal with the heat are drinking more fluids, along with wearing light clothing and staying indoors. These measures are more convenient, easier and somewhat cheaper than moving out of town to a cooler place, going swimming, using a home air-conditioner or seeking an air-conditioned location. It is important in developing regions such as Tibet to take into account cost, convenience, accessibility to adaptive facilities, and the common behaviours of local people when developing and implementing education programs. Finding out about these enabling factors may require in-depth field studies. Moreover, given the demographic differences in behaviour that showed up in our survey, it is equally important to develop specific coping instructions in relation to those at most risks to heat including babies, the elderly, outdoor workers, and people with medical conditions. As expected, a correlation between behaviour changes and perceived risk is observed in our study, and this has been identified by previous research as well [11]. Those who reported not modifying their behaviour on hot days were prone to perceive themselves as immune to the heat and have low or no sense of worry about rising temperatures in Lhasa.

Populations living in urban areas where temperatures tend to be higher than in suburban and rural locations are at greater risk of heat stress. Lack of adequate social and material resources further adds to their vulnerability. Harlan et al. [16] evaluated residents’ vulnerability to thermal environments of urban neighborhoods by analyzing the relationships between outdoor human thermal comfort index (HTCI), population characteristics and coping resources. They reported that neighborhoods with high population density, sparse vegetation and no open space were exposed to more extreme temperatures. Moreover, local residents, particularly low socio-economic groups, in those warmer neighborhoods, are more vulnerable due to limited resources. In order to understand whether or not people living in urban sub-districts in Lhasa face the same issues, we looked for inequalities in resources across our four study locations. Bakuo, located at the urban core, which is the most densely populated, crowded and noisy sub-district with few green and open spaces is expected to be the hottest area in Lhasa. Bakuo may also be most vulnerable because of the higher proportions of poor people and old dwellings with poor ventilation, small living spaces and low comfort level. The prevalence of home air conditioning is low in urban Lhasa, and lowest of all in Bakuo. We note that air conditioning at home was not found to be a strong protector against heat-related illness in our survey, and this is consistent with at least one previous study [13].

However, the strongest neighborhood social ties were seen in Bakuo sub-district. The great majority of respondents reported having a close relationship with their neighbors. Other studies in developed countries found that people on a low income suffer from poorer health than richer groups in a society because of either material poverty or a lack of social relationships and support [28-30], while our results indicated the neighbour bonding was weakest in those upper-income people. A possible explanation is that relatively well-off people are prone to isolate themselves from others in their neighborhood and make their social connections with people of similar socio-economic status in other parts of the city, or further afield. Importantly, Perry and Lindell [31] note that opinions based on personal experience of natural disaster tend to be enhanced through neighbors, friends and strong social networks. The strong neighbour ties that we observed in all sub-districts in Lhasa probably serve to promote information exchange and may be one explanation for the widespread perception of risk due to heat and the high level of reported behaviour changes. Again, for developing regions including Tibet, this indicates a heat adaptation program may be most effective in communities where people have a sense of competence and good neighbor bonding.

Apart from close relationships among neighbors, strong family connections were also observed in Lhasa. Social isolation or living alone, which is identified as a high risk factor in heat events [23], is not common in Tibet. Most of the households in this study included two or three generations, with an average size of four persons. Unlike other Chinese inland cities with a rapid increasing number of elderly persons living alone, old people in Lhasa normally live with their children and help to look after grandchildren. However, the small proportion (9.4%) of single person households in this study may be an under-estimate, since older people with chronic disease or other conditions that restrict their mobility are less likely to come out and participate in surveys. A more in-depth understanding of local circumstances and vulnerable populations is most important for developing practical adaptive measures and prioritizing actions.

Several limitations of the study should be noted. The study was conducted on a convenience basis due to local factors that prevented strictly random sampling. Older individuals and other subjects at high risk may be underrepresented in our sample. The study was only conducted within four urban sub-districts in Lhasa, so caution should be applied in generalizing the results to other parts of Tibet, particularly rural and remote areas. Additionally, as with all studies of this kind, the answers may be affected by a social desirability bias, meaning that participants are prone to report what they think to be the socially accepted answers, not necessarily their real perceptions.

## Conclusions

We recommend either a regional plan or targeted programs to address public health implications of heat or other climate extremes in Tibet, China. Our study suggests that actions are needed now to minimize the downside effects of rapid warming in Tibet, especially in urban areas, because of increasing human exposure to summer higher temperatures and uneven distribution of the resources needed to cope. There are important opportunities for local governments act on risks from heat. Firstly, the high levels of perceived risk show that the population believes temperatures are rising and are likely to affect livelihoods and health. This is a firm basis for public health surveillance to monitor heat-related conditions, paying particular attention to vulnerable groups, so that targeted interventions can be appropriately initiated and prioritized. Other steps to reduce heat stress include upgrading infrastructure in communities, improving the quality of existing old dwellings and increasing public green spaces and parks. Lastly, it is a positive sign that the majority of respondents reported that they wanted to obtain more information on heat and health. This indicates an opportunity for education programs to provide practical tips and information to better prepare for and cope with high temperatures.

## Abbreviations

IPCC: Intergovernmental panel on climate change; CDC: Center for disease control and prevention; CMS: Chronic mountain sickness; HTCI: Human thermal comfort index.

## Competing interests

The authors declare that they have no competing interests.

## Authors’ contributions

LB, QL and AW designed the study and supervised the data. LB and AW analyzed the data and wrote the paper. C, P, D, X, B and D contributed to organize field works, data collection and interpret the results. XL, SS, FW, LZ, JX, XL, HW and BY participated in the questionnaire survey. QL had the overall supervision of the project. All authors read and approved the final manuscript.
